# Process-Induced Nanostructures on Anatase Single Crystals via Pulsed-Pressure MOCVD

**DOI:** 10.3390/ma13071668

**Published:** 2020-04-03

**Authors:** Rukmini Gorthy, Susan Krumdieck, Catherine Bishop

**Affiliations:** Department of Mechanical Engineering, College of Engineering, University of Canterbury, 20 Kirkwood Ave, Christchurch 8041, New Zealand; rukmini.gorthy@pg.canterbury.ac.nz (R.G.); catherine.bishop@canterbury.ac.nz (C.B.)

**Keywords:** anatase single crystals, process-induced nanostructures, competitive growth, pp-MOCVD

## Abstract

The recent global pandemic of COVID-19 highlights the urgent need for practical applications of anti-microbial coatings on touch-surfaces. Nanostructured TiO_2_ is a promising candidate for the passive reduction of transmission when applied to handles, push-plates and switches in hospitals. Here we report control of the nanostructure dimension of the *mille-feuille* crystal plates in anatase columnar crystals as a function of the coating thickness. This nanoplate thickness is key to achieving the large aspect ratio of surface area to migration path length. TiO_2_ solid coatings were prepared by pulsed-pressure metalorganic chemical vapor deposition (pp-MOCVD) under the same deposition temperature and mass flux, with thickness ranging from 1.3–16 μm, by varying the number of precursor pulses. SEM and STEM were used to measure the *mille-feuille* plate width which is believed to be a key functional nano-dimension for photocatalytic activity. Competitive growth produces a larger columnar crystal diameter with thickness. The question is if the nano-dimension also increases with columnar crystal size. We report that the nano-dimension increases with the film thickness, ranging from 17–42 nm. The results of this study can be used to design a coating which has co-optimized thickness for durability and nano-dimension for enhanced photocatalytic properties.

## 1. Introduction

Titanium dioxide has been of high interest for its photocatalytic properties under UV light since the discovery of the Honda–Fujishima effect in 1972 [1]. The best known application of TiO_2_ is self-cleaning glass coated with Pilkington Activ^TM^ [2]. Anatase and rutile are the most widely researched phases of TiO_2_ for photocatalytic applications. The bandgap of anatase is 3.2 eV and the bandgap of rutile is 3.0 eV. Despite the wider bandgap, anatase has high photocatalytic activity (PCA) due to higher surface-adsorption rate of hydroxyl radicals [3]. Anatase also exhibits slower charge recombination rates than rutile [4]. The majority of the studies on TiO_2_ photocatalysis investigate a combination of anatase and rutile [5].

TiO_2_ nanoparticles have higher specific surface area than bulk titania coatings. Nanoparticles also have a shorter exciton path length from the point of generation to the surface, resulting in lower electron-hole recombination rates for films less than 15 nm [6]. Carbonaceous TiO_2_ enhances the PCA in the visible spectrum [7]. Recently, many studies have reported doping TiO_2_ with noble metals and other elements to extend the bandgap [8,9,10].

### Nanostructured Materials for Coating Applications

We aim to achieve a nanostructured solid material, which would have the high active surface area and low exciton migration path of nanomaterials, but without the fabrication and handling of nanoparticles. Hashimoto et al. theorized that selective nanostructuring of TiO_2_ surfaces would improve the hydrophilicity of the materials thereby making them photocatalytically superior [11]. Nanostructured TiO_2_ materials were reported to exhibit improved performance when used as electrodes for lithium-ion batteries compared to electrodes consisting of nanocrystalline anatase [12]. Nanostructured materials such as mesoporous titania have demonstrated improved photoanodic efficiency over Degussa P25 [13]. The main challenge with making practical use of nanostructured materials is in the processing of a solid coating layer on a substrate. 

**Figure 1:**
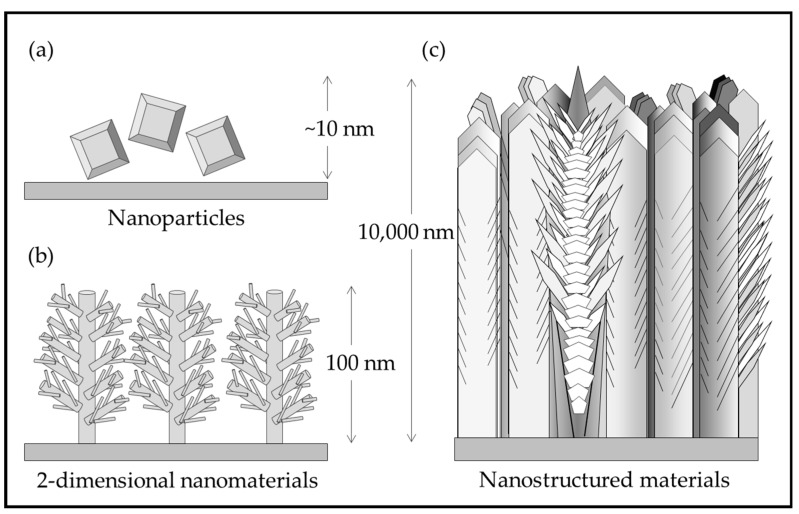
Illustration of (**a**) TiO_2_ nanoparticles; (**b**) rutile nanorods with secondary structures grown on a substrate [15]; (**c**) nanostructured TiO_2_ in a solid coating adhered to a substrate [14].

Figure 1 illustrates the differences between nanomaterials and nanostructured materials. Nanoparticles are processed in a hydrothermal solution and are difficult to attach to a substrate. Nano rods and other 2-D structures grown on substrates have not yet been demonstrated for practical coatings. The nanostructured, multiphase, thick solid coating grown by pulsed-pressure metalorganic chemical vapor deposition (pp-MOCVD) has been demonstrated to be adherent and durable [14]. 

**Figure 2:**
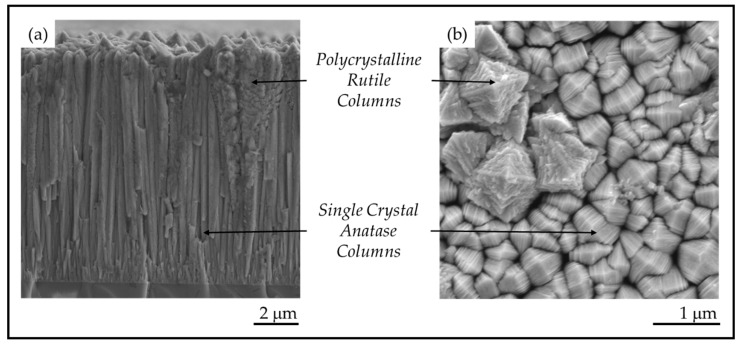
(**a**) Cross-section morphology on a fracture surface and (**b**) plan-view surface morphology of TiO_2_ films prepared by pulsed-pressure metalorganic chemical vapor deposition (pp-MOCVD).

Pulsed-pressure metalorganic chemical vapor deposition (pp-MOCVD) has been used to nanoengineer solid TiO_2_ coatings (≤17 μm thick) that are composites of anatase, rutile and carbon. The coatings shown in Figure 2 have a rarely-seen columnar morphology composed of single crystal anatase and polycrystalline rutile columns [16]. Anatase columns appear pyramidal at the top and the rough dendritic structures are polycrystalline rutile (Figure 2). The thick robust coatings produced on stainless steel substrates exhibited high antimicrobial activity under visible light [14]. Thinner films on fused silica substrates have formal quantum efficiency 59 times higher than the commercial photocatalyst Pilkington Activ^TM^ [17], measured with stearic acid degradation in UV light [18]. In previous work, we used ASTAR^TM^ analysis of TEM samples of TiO_2_ films grown on stainless steel to determine that the anatase columns were single crystals, even though they are made up of nano-plates, with A[220] columnar growth direction [14]. The properties of photocatalytic materials depend on the crystallography and morphology, but there has been no research reporting a study of nanoscale feature size in TiO_2_ bulk or coating materials to date.

In this study, coatings of TiO_2_ were produced on glass and stainless-steel substrates. All crystals exhibited the characteristic anatase *mille-feuille* and rutile *strobili* nanostructures shown in Figure 2. The thickness of the plate-like *mille-feuille* structures was observed to depend on the coating thickness, which is controlled by the number of precursor pulses. The thickness of the *mille-feuille* plates in the anatase columns is of great interest because the PCA depends significantly on the nanostructure dimension and the total surface area. The competitive columnar growth of TiO_2_ anatase single crystals and the observed relation to nanostructure dimensions were investigated.

## 2. Experimental Details

### 2.1. Pulsed-Pressure MOCVD Technology

The pp-MOCVD technique is a one-step deposition process that was developed by Krumdieck et al. to coat large objects such as turbine blades with thermal barrier coatings [19]. The process works by direct injection, at timed intervals, of metered volumes of precursor solution via an ultrasonic atomizer into a continuously evacuated deposition chamber. The flash evaporation of atomized liquid in the evacuated reactor chamber produces a sharp pressure spick, and produces a well-mixed reactor condition enabling the coating of complex shaped objects. The objects or substrates are placed on a susceptor that is heated with a water-cooled Cu induction coil. The Titanium tetra-isopropoxide (TTIP) precursor is decomposed when it encounters the heated substrate and forms a macroscopically uniform coating. Solvent and reactant product vapors are evacuated into a liquid N_2_ cold trap. At high temperatures (>500 °C) the reactor works in the mass-transport controlled regime with high precursor-arrival rate to the substrate surface during the peak of the pressure pulse [20]. The pulsed-pressure cycle is typically 6 seconds with less than 0.5 seconds of pressure rise, and 5 seconds of pump-down [21]. The pp-MOCVD process reduces the reactor and substrate geometry effects on the deposition, making it a versatile technique to coat 3D objects. 

### 2.2. Materials and Chemicals

TiO_2_ coatings were deposited on 25 mm × 25 mm × 1 mm fused silica substrates (Esco Optics, Oak Ridge, NJ, USA) and a 340 stainless steel substrate (Aperam S.A., Isbergues, France) using a TTIP precursor solution. The precursor is a dilute mixture of 5 mol% of TTIP (>97% Sigma Aldrich, St, Louis, MO, USA) in dry, HPLC-grade toluene with no carrier gas and no additional oxidizing agents. 

Table 1 provides a list of samples with their identities (IDs) and the respective number of pulses. All the samples were deposited at 525 °C for fused silica substrates and 500 °C for stainless steel substrates. The material characterizations were carried out on 6 samples prepared on fused silica substrates with the number of pulses ranging from 150 to 1000. Fused silica is snapped to provide SEM analysis of the fracture surface and measurement of the thickness. Sample G was deposited on stainless-steel 304 with 600 pulses and characterized by focused ion beam (FIB). 

### 2.3. Characterization Methods

The plan-view and fracture surface morphologies of the TiO_2_ coatings were observed using a JEOL 7000F Scanning Electron Microscope (SEM, UC, Christchurch, NZ). The samples were scored on the uncoated side using a diamond-tipped scribe and fractured into four sections to expose the cross-section of the coatings. Prior to imaging, the fractured samples were sputtered with Cr using a Quorum Tech rotary pumped coater (UC, Christchurch, NZ). Ten measurements of the film thicknesses were obtained and the mean film thickness (*w*) reported. The mean growth-rate (*GR*) of a coating prepared with *N* pulses was calculated using

(1) $$GR=\frac{w}{N}$$

The mean anatase column diameter was determined from the plan-view SEM images using the ASTM E112 standard circle-intercept method [22]. Five test circles were used to obtain the column size for each sample.

The phase composition of the coatings was determined using a Rigaku SmartLab X-ray diffractometer (XRD, UC, Christchurch, NZ) equipped with a Cu $K_{\alpha }$(λ = 1.5148 Å) source. The as-deposited samples were mounted on a flat sample holder and the detector was set up to collect from 5° to 90° in 2θ at a scan rate of 10° per minute. The spectral peaks were indexed using the RRUFF database [23].

The chemical composition of the TiO_2_ samples was analyzed using Surface Enhanced Raman Spectroscopy (SERS. The Raman spectra were obtained using a HORIBA Jobin-Yvon LabRam spectrometer (MacDiarmid Inst./GNS, Wellington, NZ) equipped with an Ar ion (514 nm) laser). The power of the laser was set at 420 μW and the sample surfaces were analyzed as-deposited. The spectra were deconvoluted using a Gaussian peak-fitting algorithm in Origin Pro software (OriginLab, Northampton, MA, USA). 

The cross section of sample G was studied using a JEOL 300CF Scanning Transmission Electron Microscope (STEM, IMRI, UC-Irvine, Irvine, CA, USA). The STEM is equipped with a 300 kV cold field emission gun and has a resolution of 80 pm. The sample for STEM imaging was prepared using a FEI Quanta 3D focused ion beam (FIB)-SEM (IMRI, UC-Irvine, Irvine, CA, USA).

### 2.4. Nanostructure Dimension Measurement

**Figure 3:**
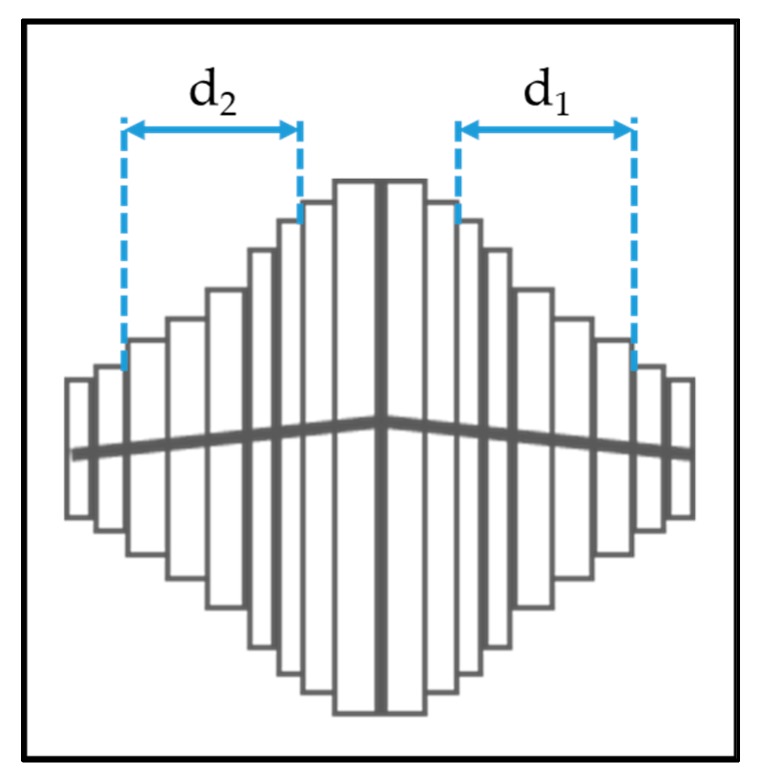
Methods to determine the nanoplate thickness for anatase columns.

The anatase columns are composed of nanoscale plates. We used a straight-forward measurement technique using the plan-view SEM images to determine the thickness of the plates. Fifteen crystals were selected from the SEM image that were symmetrical and had the most plates visible. The central plates at the peak of the crystals are always slightly thicker, so 3–5 plates as shown in Figure 3 were measured using the GMS Digital Micrograph Suite [24].

Two measurements were taken for each crystal column as shown in Figure 3. If *‘n’* plates are measured on each side of the central plates, then the thickness of a single nanoplate *‘t’* is calculated as

(2) t=12n(d1+d2)

The measurements are statistically analyzed to obtain the thickness of the nanoscale plates on the anatase columns and the measurement accuracy. This measurement is referred to as the nano-dimension.

## 3. Results

### 3.1. Phase and Composition of TiO_2_ Coatings Prepared by pp-MOCVD

**Figure 4:**
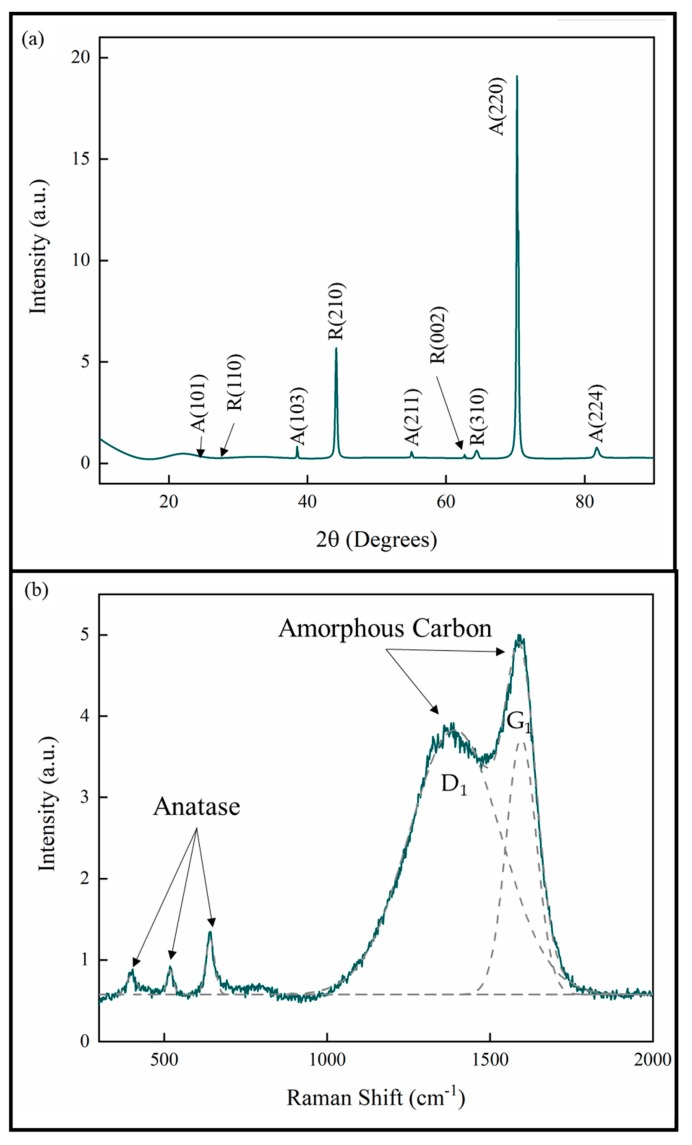
(**a**) XRD pattern and (**b**) Raman spectrum of TiO_2_ coatings prepared by pp-MOCVD.

The XRD analysis of the coatings showed that all the coatings were TiO_2_ with both anatase and rutile phases. The lattice parameters are consistent with stoichiometric TiO_2_ [25,26]. The XRD pattern given in Figure 4a is representative of all the measurements for the samples in this study. The pattern also shows that the anatase phase exhibits a strong [220] growth texture. This is consistent with previous studies of pp-MOCVD films [14,18,20,27].

A Raman spectrum representative of all the samples in this study is provided in Figure 4b. The deconvoluted peaks correspond to the anatase phase of TiO_2_ and amorphous carbon present in the films [23,28,29]. The amorphous C peak D_1_ represents aromatic carbon rings and the peak G_1_ represents the sp^2^ C=C bonds. No evidence for Ti-C was detected by Raman spectroscopy and no bulk titanium carbide phases were identified in XRD. These results are consistent with our previous work, where a uniform distribution of carbon was measured through the films thickness [27].

### 3.2. Plan-View Surface Morphologies of TiO_2_ Coatings

**Figure 5:**
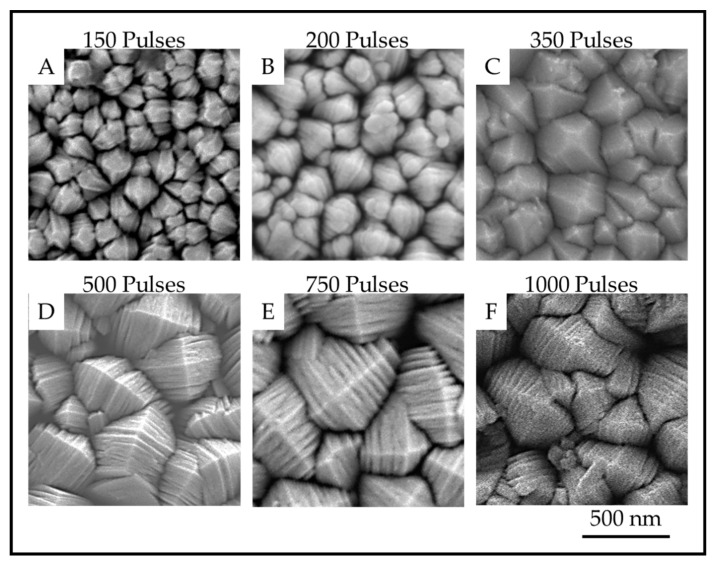
Plan-view SEM images showing the surface of anatase crystals for TiO_2_ samples on fused silica prepared with (**A**). 150 pulses; (**B**). 200 pulses; (**C**). 350 pulses; (**D**). 500 pulses; (**E**). 750 pulses and (**F**). 1000 pulses.

**Figure 6:**
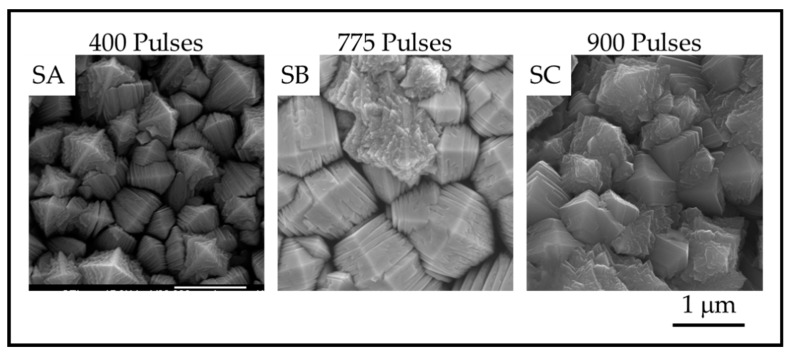
Plan-View SEM images showing the surface of anatase crystals for TiO_2_ samples on stainless steel prepared with SA. 400 pulses; SB. 735 pulses and SC. 909 pulses.

The TiO_2_ coatings were all macroscopically uniform, adherent and black in color. The SEM images of the coatings showed that the coatings were composed of columnar crystals with two distinct morphologies, anatase *mille-feuille* and rutile *strobili* as described in previous work [14]. The top of an individual anatase column resembles a plated pyramid-like structure. Figure 5 and Figure 6 show the plan-view surface SEMs of the samples deposited on fused silica and stainless steel substrates. The figures show that the surface morphologies for TiO_2_ coatings on stainless steel and fused silica substrates are similar.

The images show that as the number of pulses, and thus the thickness, increases, the number of nanoscale plates and the column diameter also increase. The thickness of the plates is not obviously different, but the measurements reveal a definite trend in nanoscale dimension. The column diameters and the surface-nanostructure dimensions for samples A-F are provided in Table 2. 

### 3.3. Analysis of Growth and Nanoscale Dimensions

**Figure 7:**
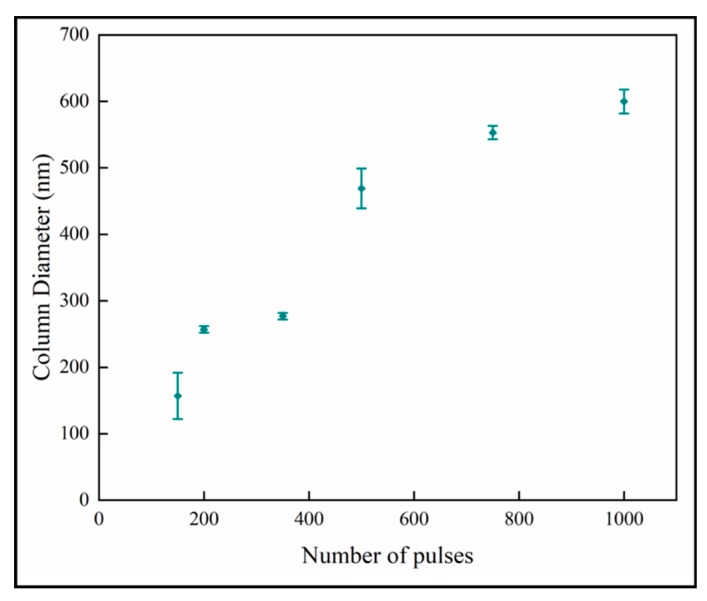
Variation of column diameter with number of pulses.

Figure 7 shows the variation of the column diameters with number of pulses. It is observed that the column diameter increases with increasing numbers of pulses. The relationship appears to have two linear regions with a break point around 600 pulses. The linear curve fit for all the data shows that the R^2^-value is 0.89. The relation at the low number of pulses indicates that the column size of ~100 nm would not have nanoplates, and this is consistent with previous results [18]. The column diameters appear to approach a plateau in the thicker films with higher number of pulses. This behavior fits the model of competitive crystal growth [30].

**Figure 8:**
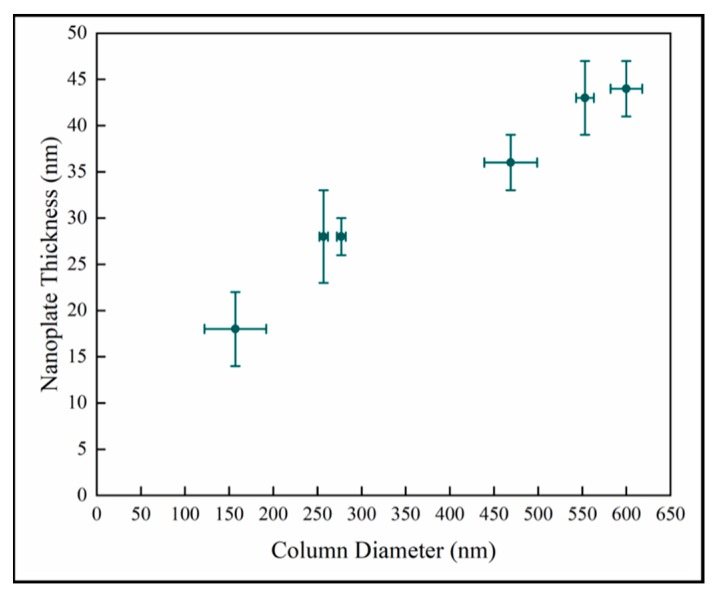
Variation of surface nanoplate thickness with column diameter.

The nanoplate thickness increases with column diameter as shown in Figure 8. We observe that there is likely a change in the relationship in the later stage as well. This is consistent with the behavior of the samples observed in Figure 7. However, this analysis depends on two difficult measurements which increases the uncertainty, and there is a wide variation in crystal diameter.

### 3.4. Fracture Surface Morphologies 

**Figure 9:**
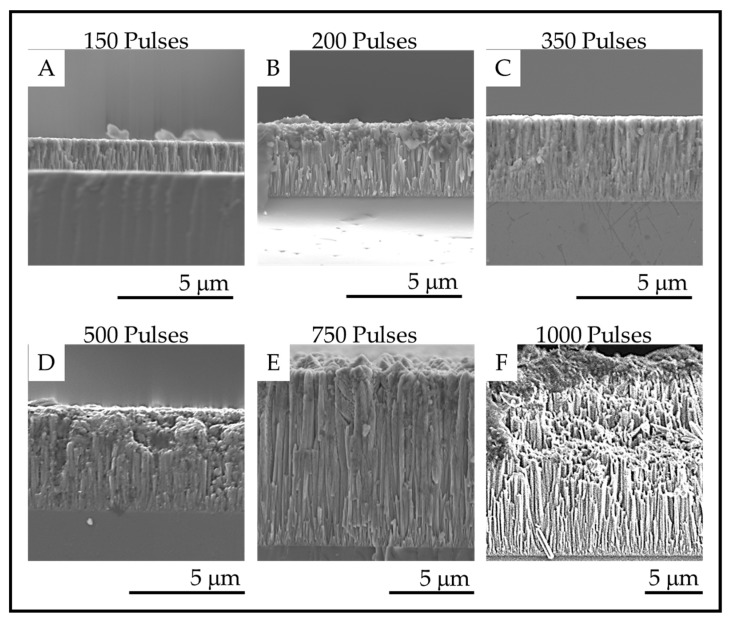
Fracture surface SEM images of the TiO_2_ coatings deposited on fused silica with (**A**). 150 pulses; (**B**). 200 pulses; (**C**). 350 pulses; (**D**). 500 pulses; (**E**). 750 pulses and (**F**). 1000 pulses.

The fracture surface images of the coatings on fused silica substrates are shown in Figure 9, and the film thicknesses are provided in the Table 3. The fracture surface SEM images show that columnar morphology extends through the entire thickness of the films. The thickness of the coatings increases with increasing numbers of pulses as expected for a mass transport-controlled deposition. 

**Figure 10:**
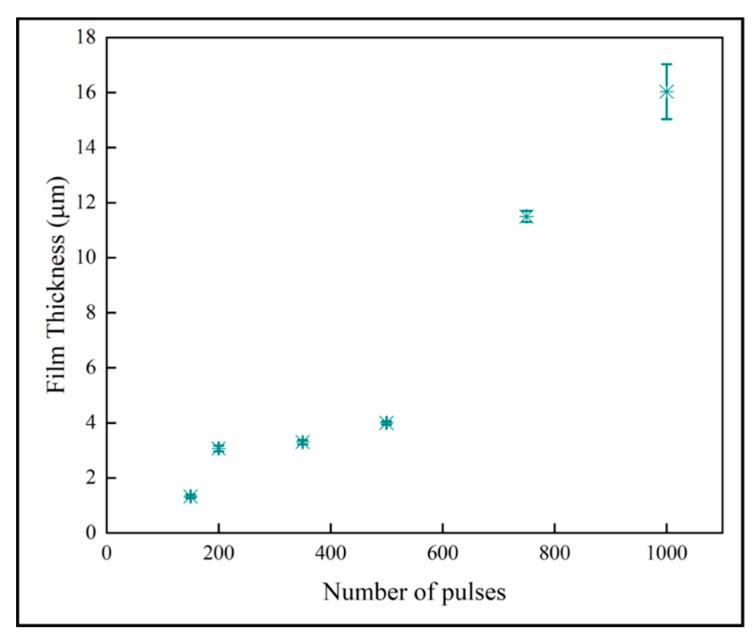
Sample thicknesses increases with number of pulses.

The variation of the film thickness with number of pulses is shown in Figure 10. The film thickness increases with the number of pulses, which is expected with mass-transport controlled growth. The thinner films below 600 pulses again seem to have a different growth regime than the thicker materials. We do not offer an explanation for this other than observing that the number of nanoplates per columnar crystal also increases dramatically from 500 pulses upward. 

**Figure 11:**
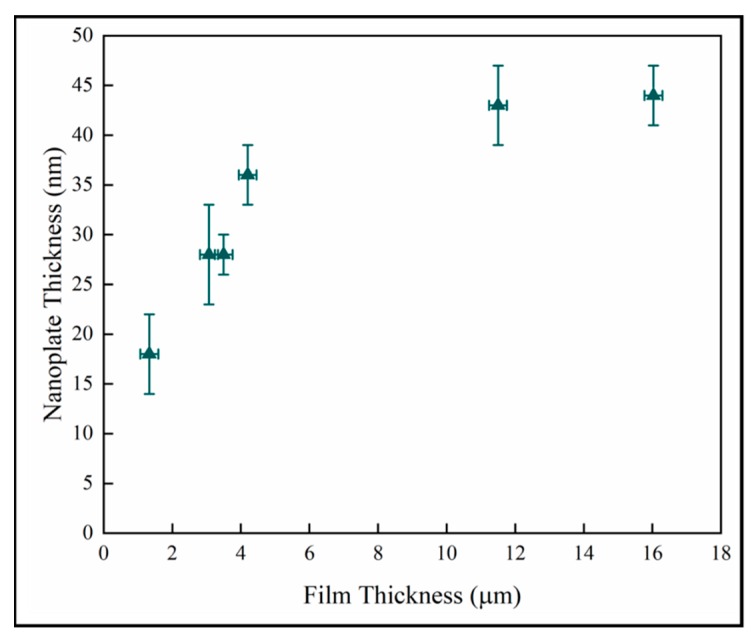
Variation of nanoplate thickness with film thickness.

Figure 11 shows the nanoplate thickness rapidly increasing with film thicknesses in the thinner coatings, but then becoming independent of thickness in the thickest films. The result for the thin samples is consistent with a previous study on initial growth of pp-MOCVD films where three growth stages were identified, early, transition and late stage growth [18].

**Figure 12:**
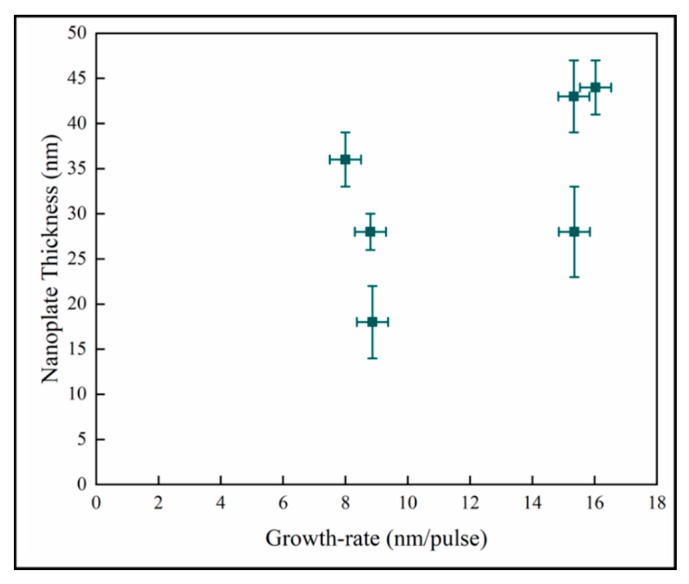
Variation of nanoplate thickness with mean film growth-rate.

In Figure 12, the variation of the nanoplate dimension with the mean film growth-rate does not demonstrate any discernable pattern between these quantities. In pp-MOCVD, as in other CVD (Chemical Vapor Deposition) processes, the growth rate is most dependent on deposition temperature. The accuracy of the measurement of temperature and the control of the induction heating means that the variability of the temperature during the majority of a deposition could be +/−15 °C. Given the Arrhenius relationship between growth rate and temperature, the variation of growth rate with pulses could essentially be no relationship, and the data represents processing variability.

### 3.5. Nanostructure Dimensions along Column Length

**Figure 13:**
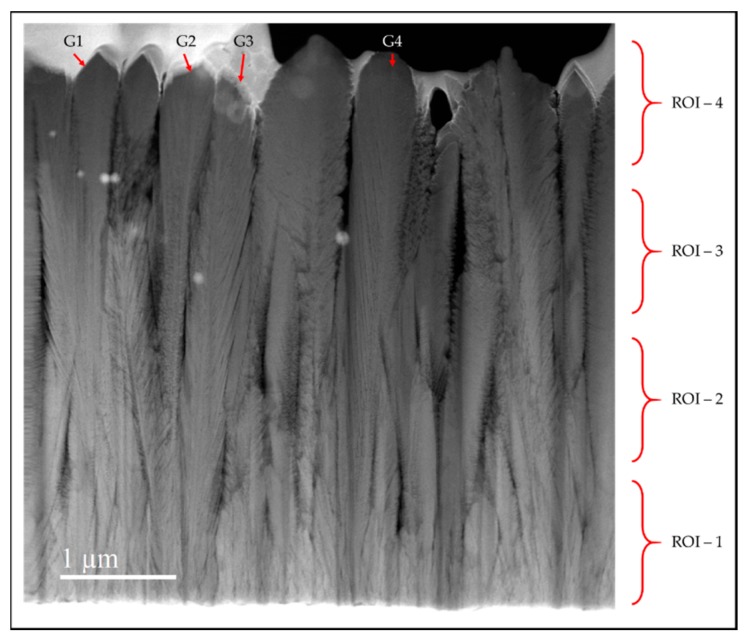
Cross-section STEM image of sample G prepared via pp-MOCVD.

Figure 13 shows the STEM image of the cross-section of sample G which is 4.77 μm thick. The white area at the base is the stainless steel substrate. The column width is much smaller at the interface of the film and substrate than at the top of the coating. Regions of Interest (ROI) are indicated which represent roughly 150 pulses of growth each. In ROI-1 near the substrate, it is harder to discern the appearance of nanoplates, and the columns have a small diameter. The columnar crystals have a tapered appearance in regions of interest (ROI) 1 and 2. ROI-2 in particular has clear evidence of the competitive growth resulting in the termination of some columns which are then overshadowed by others. The columns appear straighter in ROIs 3 and 4. This is typical of the Zone-T competitive growth [31]. 

An interesting observation here is the number of crystal columns in each region. There are higher number of crystallites observed in ROI-1 than in ROI-4 where we observe nine distinct crystal columns. This variation of the column diameters from the substrate to the top of the film provides visual evidence of competitive growth in the TiO_2_ columnar crystals during a pp-MOCVD process.

The four ROIs of 1 μm length for four anatase columns were analyzed. The anatase columns are indicated on Figure 13 by the labels G1, G2, G3 and G4. The average crystal widths from 10 measurement in each region are provided in Table 4. The column widths of the crystals at the surface of ROI-4 average ~410 nm which is consistent with the ranges of column diameters observed for the samples D and E. At the substrate-film interface in ROI-1, we observe a decrease in the dimensions of the column diameters. 

**Figure 14:**
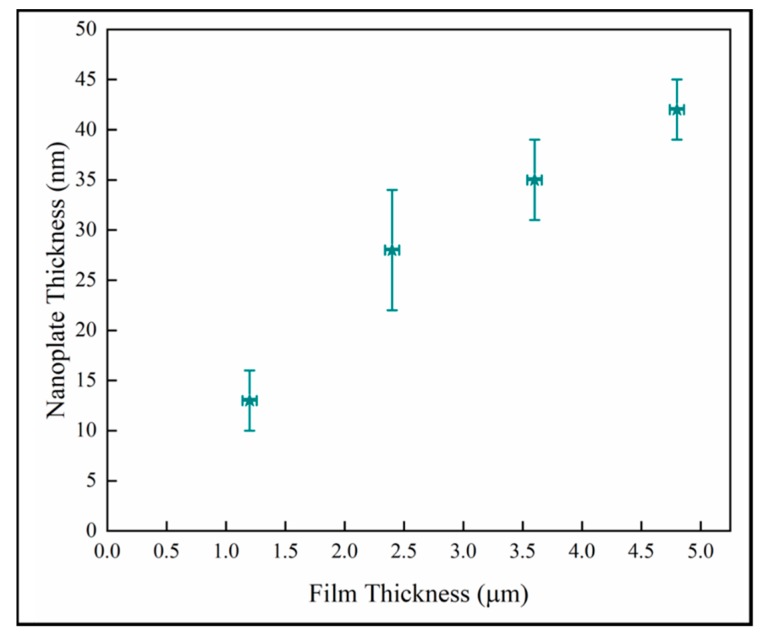
Variation of the mean nanoplate thickness with the distance from substrate in anatase column G4.

The thickness of the nanoplates for the anatase columns G4 were analyzed in the four ROIs specified in Figure 13. Figure 14 shows the variation of the nanoplate thickness plotted against the distance from substrate. The trend shows that the nanostructure thickness increases with the thickness of the films. 

## 4. Discussion

Nanostructured anatase solid coatings have been previously reported by only a few authors [32,33]. Column-like morphologies of TiO_2_ are commonly reported for rutile TiO_2_ [34] and similar structures have been reported for anatase nanocrystals [35]. Microscale plated-anatase crystals were reported from aerosol CVD [36]. Anatase columns with nanoplates resembling the ones reported in this paper were reported by one other research group who used an atmospheric pressure MOCVD and TTIP + O_2_ reactant precursor [37]. These previous studies do not report growth rate or nanostructure dimension, but from descriptions of the processing, we believe that all are relatively rapid growth conditions. High crystal growth rates have been suggested as a factor in generating unstable steps in anatase TiO_2_ [38] which could be an important mechanism in the formation of the nanoplates. The pp-MOCVD flash vaporization produces high mass flux and the temperature of 525 °C is sufficient for rapid reaction of the precursor solution.

Competitive crystal growth in columnar thin films results in increased diameter columns with film thickness and increasing crystallographic alignment [39]. The results in this study are consistent with the current theory. The range of thickness possible with pp-MOCVD allowed us to study the full range of competitive growth. The late stage plateau of crystal column diameter is clearly illustrated in the FIB-STEM sample. The diameter of crystals reaches a certain size where competition stops, but the thickness can continue to increase as crystal diameters remain the same. The orientation of TiO_2_ crystals that nucleate on the substrate is random but as the deposition progresses, the fast-growing crystal columns take over and limit the growth of the slower growing crystals. The orientation of the anatase crystals suggests the fast-growing crystals are those that have the (220) planes oriented parallel to the substrate surface [40].

**Figure 15:**
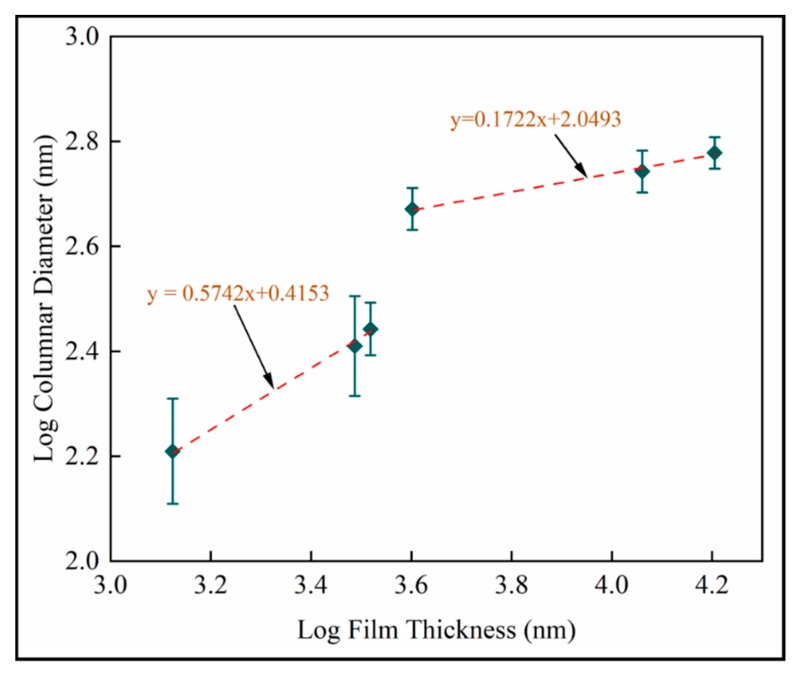
Log transformed plots of column diameter with film thickness for mean measurements on fused silica substrates (samples A–F).

Dynamic scaling theories propose power law interrelationships between microstructural features such as mound spacing, film thickness, interface width (root mean square roughness) and growth time [41]. Figure 15 is the log–log plot of column diameter with film thickness. Scaling exponents from $diameter\;\propto thickness^{p}$ are identified as p=0.57 for shorter deposition times and p=0.17 for longer deposition times. Both linear fits have $R^{2}>0.99$. The reduced exponent for thicker films supports last-stage competitive growth.

**Figure 16:**
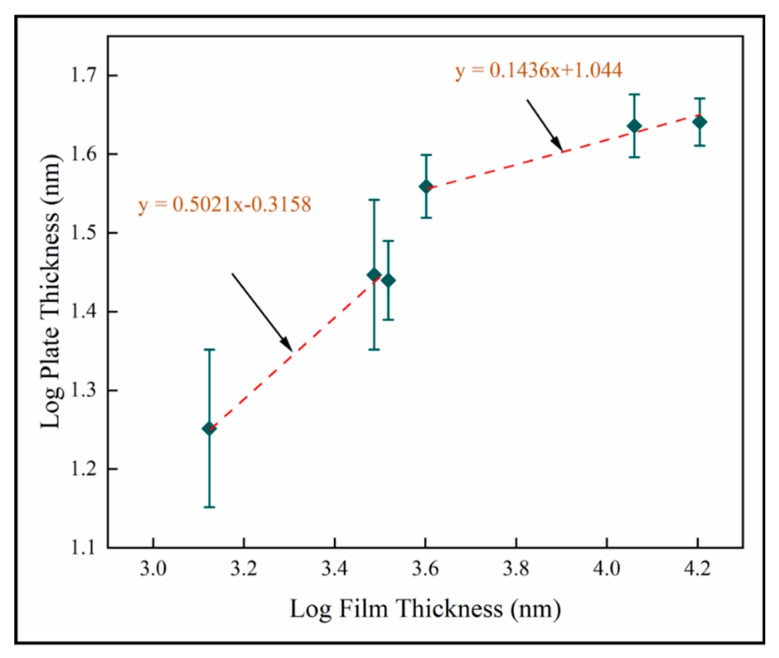
Log transformed plate thickness for mean data on fused silica substrates (samples A–F).

The nanoplate dimension is the most significant result of this study. Our objective is to enhance PCA and antimicrobial activity by inducing nanoscale structure in a solid coating. The results described in this study show that the nano-plate thickness increases with thickness and number of pulses. Figure 16 shows the log transformed plate thickness measurements with film thickness for samples A-F on fused silica. Two scaling regimes are identified from best fit lines, where $R^{2}>0.96$ for both. This shows that plate thickness increases as square root film thickness in the thinnest films investigated here. In thicker films, the plate thickness increases more slowly with film thickness. This is likely to be related to texture and supports the role of competitive growth in film development. We postulate that the increased column diameter provides more space for the nanostructures to grow and hence the resultant increase in their dimensions is observed. The nanoplate dimensions is observed to increase rapidly during the first 600 pulses, but beyond this the nano-dimension begins to plateau. We hypothesize that this is due to the relatively unstable thermodynamic conditions at a lower number of pulses compared to the conditions at a higher number of pulses. It could also be explained by the competitive columnar crystal growth. As the column diameter of the crystals increases, we also observe that each plate has feather-like nanostructures at the edges. This has not been investigated here and high resolution TEM imaging would be required to carry out a similar analysis on the dimensions of these tertiary nanostructures.

In previous work, we showed that PCA measured by photodegradation of stearic acid had a strong correlation with the surface roughness of mixed phase TiO_2_ films grown by pp-MOCVD on fused silica [18]. In that study, the two thickest films, ~1 μm and 4 μm thick, respectively, had the highest surface roughness values and the highest PCA, and correspond to samples A and D in the present work. The smallest nanoplate width, ~18 nm, was measured on sample A. The nanoplates in sample D were more than twice as thick, ~36 nm. This suggests that the PCA is not linearly correlated to the nanoplate width. Other factors could be tertiary branching, texture formation and porosity.

Another phenomenon that has not yet been studied thoroughly is the formation of multi-phase titania columns. Tavares et al. stated that the competitive growth between anatase and rutile phase TiO_2_ is dependent on the chemical kinetics that come into play during high-temperature deposition conditions. The authors also report that the quantity of O_2_ in the processing environment dictates the phase fractions of anatase and rutile [42]. As the pp-MOCVD processing takes place in a low O_2_ environment, the relatively sporadic appearance of rutile phase in these films could be explained. We have previously reported that the rutile columns grow on top of anatase crystals [14]. However, we have also observed that TiO_2_ films prepared by pp-MOCVD are composed of both anatase and rutile phases even in the early deposition stages [18]. The next stage is to study the crystallographic orientation and determine the growth conditions that favor the appearance of rutile in the films.

## 5. Conclusions

This work adds to a body of work aimed at the practical application of nano-engineered TiO_2_ solid coatings for antimicrobial touch-surfaces. The pp-MOCVD process has recently provided a processing route to produce nanostructured columnar anatase/rutile/carbon composite TiO_2_ coatings. An interesting *mille-feuille* plated nanostructure in anatase columns is investigated as one of the reasons for the observed enhancement of photocatalytic activity. The short migration path from inside the nanoplate to the large specific surface areas is key for achieving an antimicrobial coating. Surface SEM and cross-sectional STEM dimensional measurements have elucidated a relationship between the thickness of the coating and the key nano-dimension. The nano-dimension increases with the column diameter and with the thickness in a similar way as expected with competitive growth mechanisms.

The variation of the nanoplate dimension with sample thickness exhibits two trends. In the early stage depositions (≤600 pulses), the nanoplate dimension increases rapidly with the increase in the number of pulses. The samples deposited for longer durations show a weaker variability of the nanoplate dimensions with sample thickness. The variation of nanoplate dimension appears to plateau with number of pulses over 600 in the same way that column diameter approaches an apparent maximum in the thickest coatings. The relationships reported in this work can provide the basis for engineering of a practical nano-structured TiO_2_ coating with the enhanced photocatalytic activity provided by nanoengineering. The coating needs to be thick enough to have durability and long service life, but needs to be thin enough to keep the migration path length low. Because of the relationship described between column diameter in the surface SEM view and the nano-dimension, the quality control of the nano-engineered coating could be much more easily established than if nanoscale observations were required.

## Figures and Tables

**Table 1:** Sample Identifiers, Substrates and Number of Deposition Pulses.

Sample ID	Substrate	Number of Pulses
A	Fused Silica	150
B	Fused Silica	200
C	Fused Silica	350
D	Fused Silica	500
E	Fused Silica	750
F	Fused Silica	1000
G	Stainless Steel	600
SA	Stainless Steel	400
SB	Stainless Steel	735
SC	Stainless Steel	909

**Table 2:** Column diameters and nanoplate thicknesses reported as mean and standard deviation of the measurements.

ID	Number of Pulses	Column Diameter (nm)	Plate Thickness (nm)
A	150	157 ± 35	17.85 ± 3.5
B	200	257 ± 5	27.98 ± 5.3
C	350	277 ± 5	27.53 ± 2.3
D	500	469 ± 30	36.23 ± 2.7
E	750	553 ± 10	43.25 ± 3.5
F	1000	600 ± 18	43.74 ± 3.0

**Table 3:** Film thickness and mean growth-rate.

ID	Number of Pulses	Film Thickness (μm)	Growth-Rate (nm/pulse)
A	150	1.33 ± 0.03	8.87 ± 0.2
B	200	3.07 ± 0.05	15.35 ± 0.25
C	350	3.30 ± 0.04	10 ± 0.11
D	500	4.00 ± 0.03	8.4 ± 0.06
E	750	11.50 ± 0.10	15.33 ± 0.13
F	1000	16.03 ± 0.50	16.03 ± 0.5

**Table 4:** Anatase crystal column widths from sample G measured in four regions of interest (ROI) from STEM cross-section in Figure 13.

ROI	Anatase Column Width (nm)			
	G1	G2	G3	G4
1	80.7 ± 18	101.7 ± 17	221.6 ± 54	136.0 ± 30
2	92.4 ± 8	126.7 ± 31	332.3 ± 3	215.9 ± 20
3	142.4 ± 26	269.8 ± 59	418.3 ± 21	325.5 ± 49
4	315.5 ± 60	397.2 ± 53	419.7 ± 28	511.4 ± 19
